# Trends in Tuberculosis Mortality Across India: Improvements Despite the COVID-19 Pandemic

**DOI:** 10.7759/cureus.38313

**Published:** 2023-04-29

**Authors:** Karan Varshney, Hinal Patel, Shahed Kamal

**Affiliations:** 1 School of Medicine, Deakin University, Waurn Ponds, AUS; 2 Internal Medicine, Northern Hospital Epping, Epping, AUS

**Keywords:** tribal population, infectious diseases epidemiology, covid-19, people living with hiv/aids, tuberculosis

## Abstract

Introduction: The coronavirus disease 2019 (COVID-19) pandemic has had significant health implications across the globe. India is a country that has faced a double burden of COVID-19 and tuberculosis (TB) since 2020. There is a need to understand the impacts of COVID-19 on tuberculosis control programs in India. Therefore, our study aimed to determine the changes in TB mortality across India between 2019 and 2021.

Methods: In our study, we described trends in TB and COVID-19 cases reported across India. Next, we compared death totals for TB between 2019, 2020, and 2021 in India at the national and state level. We considered total TB deaths, as well as deaths by TB for tribal populations, and for those living with human immunodeficiency virus (HIV). Percent changes were calculated.

Results: In 2020, compared to 2019, there was a 15.4% decrease in TB death totals, with 28 out of India’s 36 states showing a decrease during this time period. While total deaths increased in 2021 compared to 2020, decreases did occur in 2021 compared to 2019. Deaths by TB for individuals living with HIV decreased by 16.0% across India. At a national level, there was a notable rise in TB deaths among tribal populations, though this was not universal across states.

Conclusion: While the majority of the world has seen an increase in new TB cases and TB deaths annually since the start of the COVID-19 pandemic, there have instead been decreases in India during this time period. More research is required to understand the factors that have led to this decrease in TB deaths. Furthermore, additional allocation of resources is required to better support vulnerable populations in states where TB death totals have increased, especially among tribal populations.

## Introduction

The coronavirus disease 2019 (COVID-19) pandemic has had devastating health consequences on populations all over the world. The pandemic has also been shown to have an impact on the control of other communicable diseases, such as tuberculosis (TB). Previous analysis demonstrated that there was a sharp decline in reporting of TB across high-burden countries since the start of the COVID-19 pandemic [1].

One country with a high burden of TB that has been greatly impacted by COVID-19 is India (Bharat). Currently, India has the highest burden of TB in the world [2]. However, it has been previously demonstrated that the total number of reported cases of TB across India has declined since the start of the COVID-19 pandemic; there were notable decreases in reported TB cases across India in 2020 and 2021 compared to 2019 [3]. While the number of reported cases has sharply declined in India, little is known about the implications of this on mortality. We, therefore, sought to determine the changes in TB death totals across India since the start of the COVID-19 pandemic.

## Materials and methods

For our study, we first compared trends in total TB cases, pulmonary TB cases, and extrapulmonary cases for 2019, 2020 and 2021; alongside this, we compared trends in COVID-19 case and death totals annually since the start of the pandemic. Next, we compared total TB deaths across India in 2019, 2020 and 2021. These comparisons were made at the national level, as well as at the state level for each of India’s 36 states. Thereafter, we analyzed changes in TB death totals among tribal populations, and for individuals co-infected with human immunodeficiency virus (HIV). Tribal populations were specifically chosen for our analysis as they have been shown to have a higher burden of TB compared to the general population [4]. Similarly, TB death totals among individuals co-infected with HIV were analyzed as this population has been shown to have significantly higher rates of TB mortality compared to the general population [5,6]. In our study, percent changes were calculated for state and national level data.

Data for this study was retrieved from the Government of India’s TB Reports for 2020-2023 [7-10]. The data from these reports are publicly available. The Government of India publishes TB reports annually and provides data at the state and national level regarding TB incidence, prevalence, outcomes, and drug resistance [11]. These reports are published as part of the Indian government’s National Tuberculosis Elimination Program (NTEP), which is an initiative that aims to end TB infection in India [11,12] ultimately. As well, COVID-19 case and death data for India was retrieved from Worldometer [13], which is a source that has publicly available data on COVID-19 incidence and mortality for countries across the world.

## Results

In 2019, there was a total of 2,404,815 TB cases detected across India, and this decreased by 24.9% in 2020 (1,805,670 cases). While there was a higher number of total TB cases detected in 2021 (2,135,830 cases) compared to 2020, this was 11.2% lower compared to 2019. Pulmonary cases of TB made up 73.4% of cases in 2019 (1,764,416 pulmonary cases), and this decreased to 72% of total cases in 2020 (1,291,986 pulmonary cases) and again constituted 72% of total cases in 2021 (1,528,000 pulmonary cases). A summary of changes in TB case notifications is shown in Table 1.

**Table 1:** Trends in TB Reporting in 2019, 2020 and 2021 TB: Tuberculosis

Year	Total TB cases detected	% change in total TB cases	Total pulmonary TB cases detected	% change in TB pulmonary cases	Total extra-pulmonary TB cases detected	% change in TB extra-pulmonary cases
2019	2,404,815	Reference	1,764,416	Reference	640,399	Reference
2020	1,850,670	-24.9	1,291,986	-26.8	513,684	-19.8
2021	2,135,670	-11.2	1,528,000	-13.4	607,830	-5.1

Along with the decrease in detected cases of TB in 2020 compared to 2019, there was a total of 10,267,283 reported cases of COVID-19 in India in 2020, with 152,489 deaths due to COVID-19. In 2021, India reported 24,594,296 COVID-19 cases (2.4 times higher than in 2020), and 334,792 deaths (2.2 times higher than in 2020).

In 2019, reported death totals of notified cases for TB were 89,823. In 2020, this decreased to a total of 76,002 deaths, signifying a 15.4% decrease during this time period. In this same period, at the national level there was an 18.8% decrease in TB deaths for patients treated in the public sector (69,843 deaths in 2019 and 56,705 deaths in 2020), and a 3.4% decrease in the private sector (19,980 deaths in 2019 and 19,297 deaths in 2020). Male death totals decreased by 22.6% (from 65,285 deaths in 2019 to 54,051 deaths in 2020), and female totals decreased by 10.4% (with 24,456 deaths in 2019 and 21,913 deaths in 2020).

Reported TB death totals decreased across 28 states, with the largest percent decrease being seen in Lakshadweep (100% decrease with zero deaths in 2020), Andaman and Nicobar Islands (36.0% decrease), Andhra Pradesh (30.5% decrease), and Manipur (25.7% decrease). Notably, there was a decrease of 3,496 total TB deaths in Uttar Pradesh over this time period (19.0% decrease). Total TB deaths increased in seven states, with the largest percent increases occurring in Goa (23.6% increase), Arunachal Pradesh (21.9% increase), and Jharkhand (19.3% increase). 

Across the national level, there were minimal changes in TB death totals among tribal populations in India, with 7944 deaths being reported in 2019 and 7992 deaths in 2020 (0.6% increase). In Odisha, the state with the highest number of tribal population deaths by TB, there were minimal changes in 2019 and 2020, with a 2.5% increase in TB deaths occurring over this time period. However, there was variation across numerous states, with the states of Jharkhand and Rajasthan both showing notable increases. Between 2019 and 2020, the total TB deaths among tribal populations in Jharkhand rose by 17.7% (887 deaths in 2019, and 1044 deaths in 2020), and by 33.3% in Rajasthan (541 deaths in 2019 and 721 in 2020).

In terms of populations living with HIV, there was also a drop in TB deaths at the national level. Total reported deaths due to TB among people living with HIV in India was 5388 in 2019 and 4525 in 2020; there was hence a 16.0% decrease in deaths. At the state level, the largest decreases in deaths were seen in Andhra Pradesh (28.6% decrease), and Maharashtra (28.5% decrease). Notably, some states had an upsurge in deaths. For example, there was a 271.4% increase in TB deaths for HIV patients in Chandigarh (seven deaths in 2019, and 26 deaths in 2020), and an 84.6% increase in Jharkhand (13 deaths reported in 2019, and 24 in 2020). A summary of all percent changes in 2020 compared to 2019 is shown in Table 2.

**Table 2:** Percent increase/decrease in TB deaths across India in 2020 compared to 2019 TB: Tuberculosis

Location	Percent (%) increase/decrease in 2020 compared to 2019		
	Total TB deaths	TB deaths among the HIV-positive population	TB deaths among tribal populations
India	-15.4	-16.0	0.6
Andaman and Nicobar Islands	-36	N/A	20
Andhra Pradesh	-30.5	-28.6	-20
Arunachal Pradesh	21.9	0.0	59.3
Assam	-22.7	-6.7	-17.6
Bihar	12.3	1.0	N/A
Chandigarh	13.2	271.4	N/A
Chhattisgarh	-13.9	-14.5	-14.1
Dadra and Nagar Haveli and Daman and Diu	-32	N/A	83.3
Delhi	-4.9	-5	N/A
Goa	23.6	200	N/A
Gujarat	-20.3	-18.7	4.1
Haryana	-6.1	-12.3	N/A
Himanchal Pradesh	-13.6	-16.7	-18.2
Jammu and Kashmir	4.7	-62.5	-50
Jharkhand	19.3	84.6	17.7
Karnataka	-19.9	-5.4	14.5
Kerala	-8.8	54.3	19.4
Ladakh	-17.4	N/A	-13.0
Lakshadweep	-100	N/A	-100
Madhya Pradesh	-12.6	11.5	-9.5
Maharashtra	-20.5	-28.5	-3.3
Manipur	-25.7	30	5.6
Meghalaya	-0.5	53.8	28.0
Mizoram	4.2	100	68.1
Nagaland	-10.2	54.5	0.0
Odisha	-8.5	-2.2	2.5
Puducherry	-31.0	-75	N/A
Punjab	-5.6	28.2	N/A
Rajasthan	-3.2	-25	33.3
Sikkim	0.0	N/A	-28.6
Tamil Nadu	-24.5	-22.7	-55
Telangana	-21.6	-27.2	-33.6
Tripura	-8.1	50	116.7
Uttar Pradesh	-19.0	-19.2	-10.4
Uttarakhand	-16.9	38.1	N/A
West Bengal	-20.3	-26.4	N/A

In the year 2021, there were still a fewer number of TB deaths across India compared to 2019; overall there was a 1.96% decrease in deaths in 2021 compared to 2019. However, this death total (88,060) was higher in comparison to 2020. There was a decrease in total TB deaths across 20 Indian states compared to 2019, with the largest decreases being in the Andaman and Nicobar Islands (48.0% decrease), Andhra Pradesh (38.0% decrease), and Puducherry (36.2% decrease). There were also increases in TB deaths across the remaining states during this time period, with the largest being a 143.1% increase in Goa, and a 64.1% increase in Bihar.

In terms of populations living with HIV across India, there was a 16.0% decrease in TB deaths in 2021 compared to 2019; the number of deaths in 2021 for this population was nearly identical to the number of deaths in 2020 (4525 in 2020, and 4524 in 2021). In terms of tribal populations across India, there was a 15.9% increase in TB deaths compared to 2019 (9208 deaths in 2021). While there was a decrease in deaths across eight states, there was an increase across 16 states. The largest decreases occurred in Jammu and Kashmir (62.5%) and Tamil Nadu (36.7%), whereas the largest increases occurred in Mizoram (78.7%) and Arunachal Pradesh (66.7%).

## Discussion

Our study has shown that, at a national level, there have been notable decreases in TB death totals across India since the start of the COVID-19 pandemic. Critically, this has occurred despite an increase in total TB deaths worldwide in 2020 and 2021 [14]. The World Health Organization's 2022 Global Tuberculosis Report has indicated that, across the overwhelming majority of high-burden TB countries, there was a rise in TB deaths since the start of COVID-19, and this was partially attributed to a decrease in detection of new cases [15]. While India has seen a steady decline in reported TB cases annually since the start of the COVID-19 pandemic, in consideration of the decline of total deaths due to TB in India, it is plausible that the lower rates of TB case reporting may reflect actual declines in disease incidence. Furthermore, it demonstrates that public health initiatives launched and maintained, as part of the NTEP, may have shown effectiveness in controlling TB. Examples of such initiatives include India’s TB preventive treatment regimen roll-out, cash incentive schemes to improve nutrition, and application of family-centric care models for TB [16]. However, it is crucial to denote that there were increases in TB cases and deaths in 2021 compared to 2020, and these corresponded to sharp increases in the COVID-19 burden across India. Ongoing monitoring in upcoming years is necessary to determine if continual progress is made in addressing both of these infectious diseases.

This trend regarding declines in deaths due to TB has also been shown for populations living with HIV in India, both in 2020 and 2021. Amongst tribal populations, while there has been an increase in TB deaths at a national level, this increase was relatively minimal in 2020. However, there was shown to be a sharp increase in tribal population deaths in 2021. Therefore, it is important that TB control programs across India work to ensure that they are appropriately serving tribal populations, who may be at an elevated risk of mortality. A previous study has shown that tribal populations may be more likely to delay health seeking behavior after development of TB symptoms [17]; it is not clear if this can serve as a viable explanation for the increases in TB deaths among tribal populations, or if there are other important factors that also need to be considered. More research is hence required to understand this concerning trend in TB mortality among tribal populations in India.

At the state level, there has been notable variation in TB deaths. While numerous states, such as Uttar Pradesh and Andhra Pradesh, have shown effectiveness in lowering TB death totals during the pandemic, numerous other states have instead shown increases in death tolls. Alarmingly, some Indian states have seen considerable rises in TB deaths among tribal populations and populations living with HIV. Further research is needed to investigate the factors associated with the decreases, and increases, that are unique among subpopulations at the state level.

It is imperative that national and state governments continue to prioritize supporting TB control and prevention programs alongside COVID-19 control efforts. Supporting vulnerable populations in states that have been most negatively impacted will be integral. Public health programs should focus on ensuring that testing facilities are widely available, and that individuals are supported in adhering to treatment regimens. Usage of video-directly observed therapy (vDOT), where treatment adherence is supported and monitored through the usage of video services, has had positive impacts in other contexts [18] and may offer utility in improving overall TB outcomes across India. However, more research is required to better understand how vulnerable populations can best be supported.

Alongside the findings of our work, the limitations need to be considered. It is possible that a high number of TB deaths in India may not have been accounted for due to the COVID-19 pandemic, and therefore may not have been included in the available datasets. Furthermore, our analysis did not account for patients who never received follow-up, or failed treatment (but did not immediately die). Lastly, as our study was an ecological analysis that used data at the national and state level, we cannot make conclusions regarding outcomes in specific towns and cities. Other limitations of ecological analyses that must be considered here are an inability to demonstrate causality [19], and a lack of consideration of potential confounding variables [20]. Regardless of these limitations, our study provides important insights regarding trends in TB mortality across India since the start of the COVID-19 pandemic.

## Conclusions

Our study has demonstrated that there have been notable decreases in TB cases and death totals across India since the emergence of COVID-19. This trend has occurred at the national level, though there have been notable exceptions at the state level where death totals have instead increased - this is particularly salient among tribal populations. In consideration of the enormous burden of TB in India, it is imperative that public health programs continue to control the spread of both TB and COVID-19 concurrently.
